# Advanced arithmetic optimization algorithm for solving mechanical engineering design problems

**DOI:** 10.1371/journal.pone.0255703

**Published:** 2021-08-24

**Authors:** Jeffrey O. Agushaka, Absalom E. Ezugwu

**Affiliations:** 1 School of Mathematics, Statistics, and Computer Science, University of KwaZulu-Natal, Pietermaritzburg, KwaZulu-Natal, South Africa; 2 Department of Computer Science, Federal University of Lafia, Lafia, Nasarawa State, Nigeria; Torrens University Australia, AUSTRALIA

## Abstract

The distributive power of the arithmetic operators: multiplication, division, addition, and subtraction, gives the arithmetic optimization algorithm (AOA) its unique ability to find the global optimum for optimization problems used to test its performance. Several other mathematical operators exist with the same or better distributive properties, which can be exploited to enhance the performance of the newly proposed AOA. In this paper, we propose an improved version of the AOA called nAOA algorithm, which uses the high-density values that the natural logarithm and exponential operators can generate, to enhance the exploratory ability of the AOA. The addition and subtraction operators carry out the exploitation. The candidate solutions are initialized using the beta distribution, and the random variables and adaptations used in the algorithm have beta distribution. We test the performance of the proposed nAOA with 30 benchmark functions (20 classical and 10 composite test functions) and three engineering design benchmarks. The performance of nAOA is compared with the original AOA and nine other state-of-the-art algorithms. The nAOA shows efficient performance for the benchmark functions and was second only to GWO for the welded beam design (WBD), compression spring design (CSD), and pressure vessel design (PVD).

## 1. Introduction

Optimization techniques are popular for solving real-world problems. Finding solution to these complex, nonlinear, and multimodal real-world problems usually requires reliable optimization techniques, such as metaheuristic algorithms, which have proved to be a reliable optimization technique for such problems. The popularity of metaheuristic algorithms hinges on their ease of use and implementation, their being gradient-free, and having the ability to by-pass local optima. Metaheuristic algorithms have been successfully applied to solve problems in engineering, medicine, and many other areas.

Nature has inspired many metaheuristic algorithms; they solve optimization problems by mimicking natural phenomena. These phenomena cover a range of natural processes from such areas as biology, physics, chemistry and swarms (population-based) [1,2]. The bio-inspired metaheuristic algorithms are frequently inspired by the laws of natural evolution. The randomly generated search agents are evolved by combining the best individuals after every iteration during the search process. Examples of these bio-inspired metaheuristic algorithms include genetic algorithms (GA) [3], the artificial algae algorithm (AAA) [4], and the evolution strategy (ES) [5]. The physics- and chemistry-based based metaheuristic algorithms mimic some physical rules in the universe, for example the simulated annealing (SA) [6], gravitational search algorithm (GSA) [7,8], and the artificial chemical reaction optimization algorithm (ACROA) [8]. The swarm-based algorithms are population-based; they mimic the social behavior of animals in groups. Popular swarm algorithms include the particle swarm optimization (PSO) [9] and the ant colony optimization (ACO) [10].

The arithmetic optimization algorithm (AOA) is a recently proposed population-based metaheuristic algorithm. The algorithm is based on the distributive behavior of the arithmetic operators of multiplication (M), division (D), subtraction (S), and addition (A). The performance of the AOA was investigated using twenty-three benchmark functions, six hybrid composite functions, and several real-world engineering design problems. The AOA experimental results showed promising results when compared against results from eleven other well-known optimization algorithms [11].

The distributive power of the arithmetic operators gives the AOA its unique ability to find the global optima for optimization problems used to test its performance. However, several other mathematical operators exist, which have the same or better distributive properties, which could be exploited to enhance the performance of AOA. This motivated us to use the high-density values that the natural logarithm and exponential operators can generate to enhance the exploratory ability of AOA. The addition and subtraction operators are still used for exploitation. The major contribution of our work can be summarized as follows:

- We propose a new advanced arithmetic optimization algorithm which we refer to as the nAOA.
- The nAOA improves the exploratory ability of original AOA by using the high-density numbers generated by the natural logarithm and exponential operators.
- The candidate solutions are initialized using the beta distribution instead of the default random number initialization scheme.
- The random variables and adaptations used in the algorithm have beta distribution.

The rest of the paper is organized as follows. In Section 2, the literature is review and discussed. We presented the proposed algorithm in Section 3. Section 4 covers the experimental setup, results, and discussion. Finally, Section 5 presents the concluding remarks and suggests future research directions.

## 2. Literature review

The sine cosine algorithm (SCA) uses a mathematical model based on sine and cosine functions to achieve an optimal solution [12]. Research results proved the algorithm’s ability to explore different search space regions, to avoid being stuck in local optima, and to converge towards the global optimum. Furthermore, the SCA algorithm showed promising abilities in solving real-world problems by obtaining a smooth shape for the airfoil problem with very low drag.

A comparative study of recent algorithms, including the arithmetic optimization algorithm (AOA), the salp swarm algorithm (SSA), the slime mould optimization algorithm (SMA), and the marine predators algorithm, was carried out [13]. Based on the study, a new hybrid of the slime mould algorithm and the simulated annealing algorithm (HSMA-SA) was proposed to strengthen the exploitation and exploration abilities of the hybrid algorithm. The hybrid was applied to structural engineering design problems, where it showed promising results.

The arithmetic optimization algorithm was used to boost the artificial neural network in the proposed (IANN-AOA) where it was applied in solving the damage quantification problem [14]. The main idea is for the improved indicator to eliminate the healthy elements from the numerical model. The data for the damaged elements collected from an improved indicator’s damage index is used as input with the damage level as output. The results for the IANN-AOA showed that the damaged elements are predicted with higher precision by the improved indicator. The result is the same for damage quantification, but the results for IANN-AOA are more accurate than those for IANN-BCMO.

Premjumar et al. [15] proposed the multi-objective version of the arithmetic optimization algorithm (MOAOA). The algorithm was used for solving real-world constrained multi-objective optimization problems (RWMOPs) found in mechanical engineering, chemical engineering processes and syntheses, and power electronics systems. The performance of the MOAOA was tested on a set of 35 constrained RWMOPs and five ZDT unconstrained problems and compared with four other state-of-the-art multi-objective algorithms. The superiority of the MOAOA over the other algorithms considered is confirmed by its high accuracy and coverage across all objectives [15].

An improved arithmetic optimization algorithm (dAOA) was proposed, which used a modified version of the extreme learning machine (ELM) model for the identification of the proton exchange membrane fuel cells (PEMFCs) [16]. The configurations of the ELM were optimized by the improved algorithm, which in turn minimized the sum of the square error between the output voltage of the real PEMFC data and the output voltage. Their simulation showed that the proposed dAOA provided accurate parameters of the PEMFC stack system.

## 3. The proposed nAOA

In our proposed improvements for the AOA, the optimization process starts with initializing the candidate solutions using the beta distribution. This was chosen because so many authors have used many other distributions besides the random number to generate the initial population, with varying levels of success [17–20]. The candidate solutions are improved after every iteration according to the optimization rules. Stochastic processes are used to find optimal solutions, so the probability of getting the optimal solution increases with multiple runs.

The optimization process goes through two phases: exploration and exploitation. Exploration refers to scouring a new area/region within the search space for an optimal solution, whereas exploitation refers to scouring the neighborhood of already visited areas for the optimal solution. A good balance between exploration and exploitation can guarantee an optimal solution. For our proposed nAOA, the natural log (L ’*ln’*) and the exponential (E ’*e’*) operators are used to achieve the exploration, while the addition (A ’*+’*) and subtraction (S ’*-*’) operators are used to achieve the exploitation.

### 3.1. Motivation

**Fig 1:**
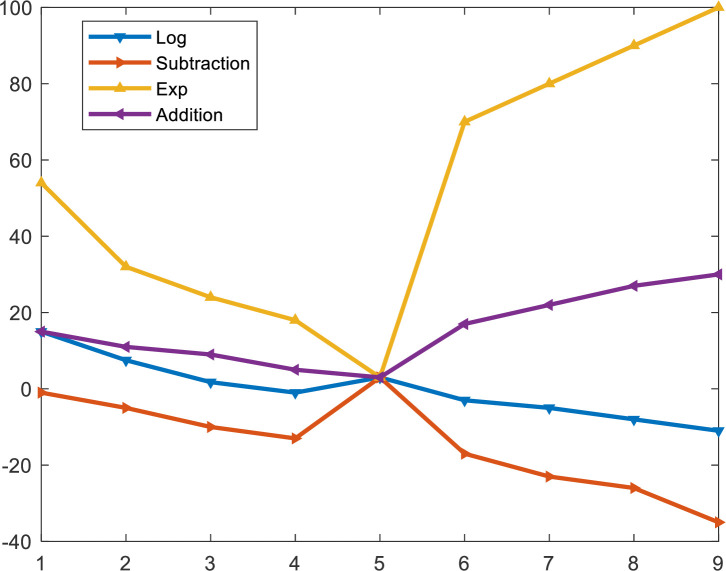
Effect of the optimization operators.

Arithmetic is an elementary branch of mathematics, and one of the oldest. It deals with the study of numbers and properties of operators applied to them. The traditional operators are addition, subtraction, division, and multiplication. However, it also involves advanced operators like logarithmic functions, exponentiation, computation of percentages, and square roots [21]. Abualigah et al. [11] used addition, subtraction, division, and multiplication for optimization in AOA. The success of AOA as an optimizer greatly motivated us to consider using other advanced arithmetic operators for our proposed nAOA. The logarithm and exponential functions are used at the exploration phase to update the candidate solutions and the addition and subtraction are used for the exploitation phase. The behavior of the optimization operators during the optimization process is shown in Fig 1.

### 3.2. Optimization process

**Fig 2:**
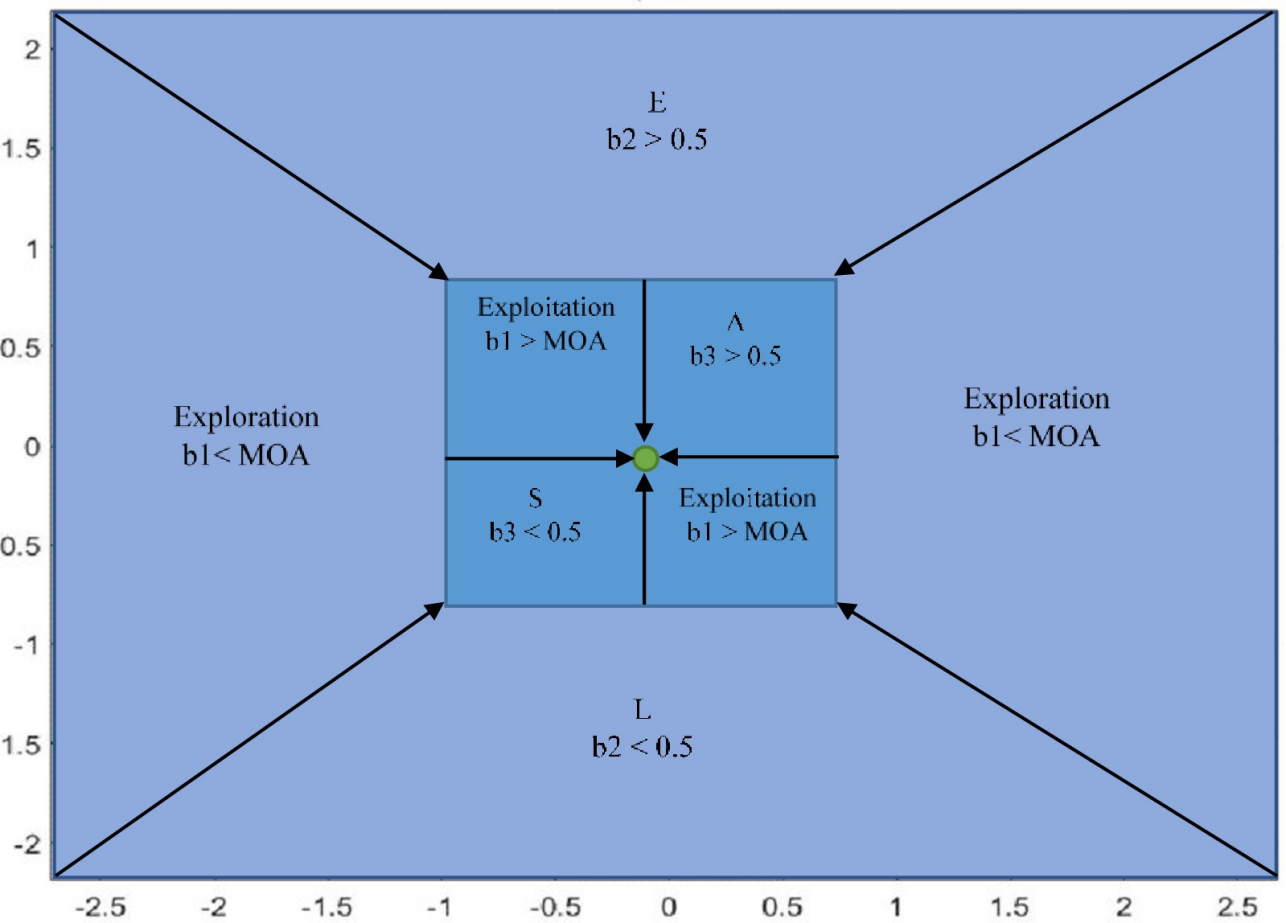
Exploration and exploitation phases of nAOA.

After the candidate solutions have been initialized, the optimizer needs to decide into which optimization phase to go. The value of the math optimization accelerator (MOA) function, defined in Eq 1, determines that phase. The exploration phases are shown in Fig 2, as used by our proposed algorithm. A detailed description of the phases is given in the next subsection.

$$MOA(C_{i})=Min+C_{i}\times \left(\frac{Max-Min}{M\_iter}\right)$$ (1)

where *C*_*i*_ is the current iteration, *Max*, *Min* are, respectively, the maximum and minimum values of the accelerator function, *M*_*iter* is the maximum number of iterations, and *MOA*(*C*_*i*_) is the value of the accelerator function at the *i*^*th*^ iteration.

The exponential function is everywhere continuous and increasing. It is asymptotic around the x-axis. It is one-to-one, and it can be shown to be mapped onto R+. The logarithmic function is the inverse of the exponential function, it is also continuous and increasing everywhere. The exponential and logarithmic functions’ ranges are used to set the directions given in Fig 1, which greatly influences the exploration ability of the proposed nAOA.

### 3.3. Exploration phase

The value of MOA is compared with a randomly generated beta distributed number (*b*1); this determines the phase nAOA goes into. Exploration in nAOA is carried out by the natural log and the exponential operators. The behavior of the two operators can be seen in Fig 1. The candidate solutions are updated using these two operators at this phase. The high dispersion of values generated by the operators makes them ideal for exploration. They can search new regions in the search space for an optimal solution; however, they are unable to converge to the optimal solution, unlike the addition and subtraction operators. In essence, the *ln* and *e* operators are complementary.

**Fig 3:**
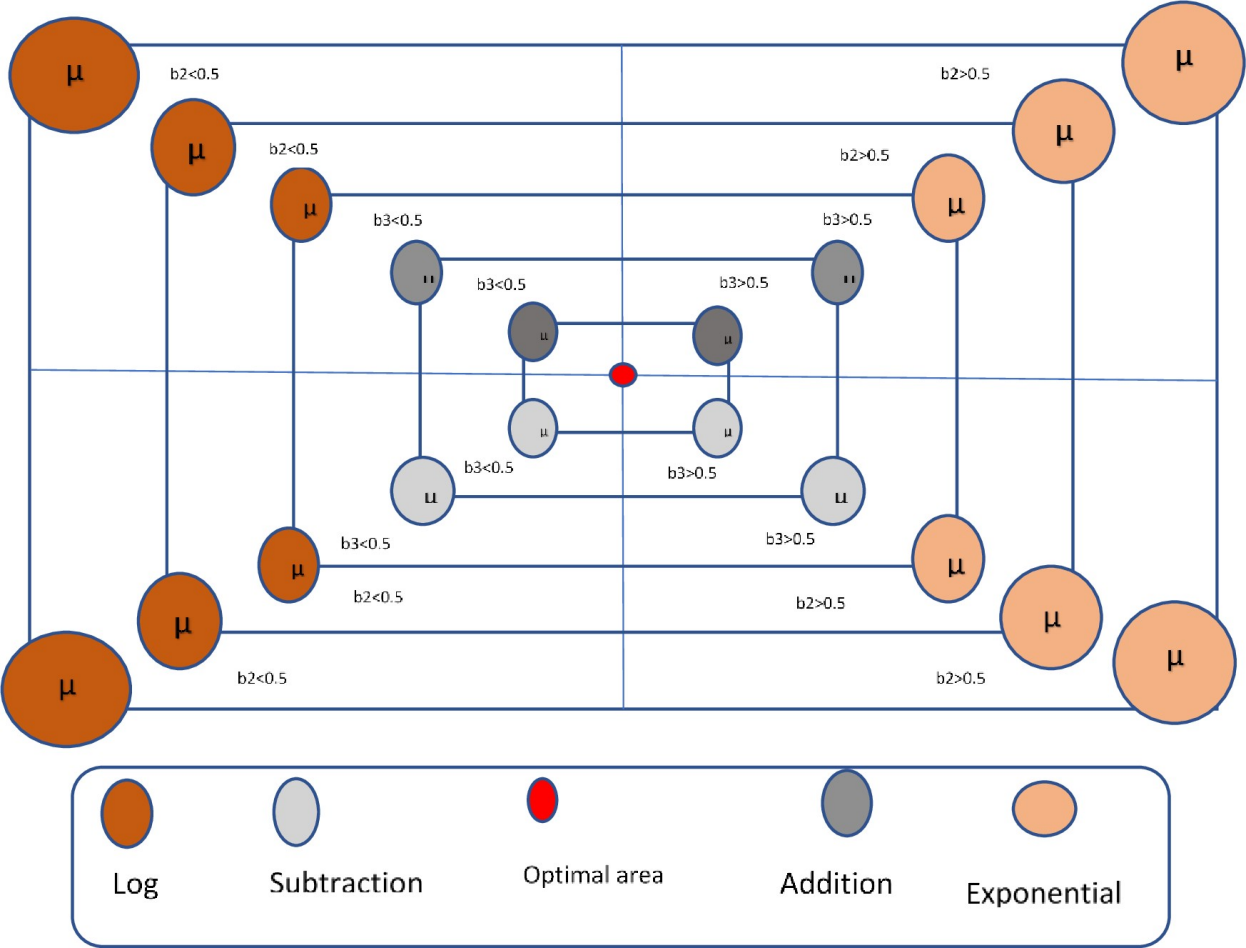
The process of updating the candidate solutions.

The nAOA exploration phase is based on the model given in Eq 2, given below. If *b*1<*MOA*, the exploration phase is activated, executing either the "*ln"* or the *e* operator. A second beta distributed random number (*b*2) is generated, if *b*2<0.5, the *ln* operator is executed. While the *ln* operator is executing, the *e* operator is ignored. If *b*2≥0.5, the *e* operator is executed, while also ignoring the *ln* operator. We used a stochastic scaling coefficient (*μ*) to increase the diversity of the exponential or logarithmic values so as to explore more diverse regions of the search space. This helps nAOA avoid getting stuck in local optima. Fig 3 is a model of how the candidate solutions are updated using the simplest arithmetic rule as shown in Eq 2. The math optimization probability (MOP) is given in Eq 3.

$$X_{new}(i,j)=\left\{ \begin{array}{c} best(j)\log\left(abs(\left(MOP+\epsilon )\times (({UB}_{j}-{LB}_{j})\times \mu +{LB}_{j}\right))\right),b2<0.5\\ best(j)\exp(MOP+\epsilon )\times (({UB}_{j}-{LB}_{j})\times \mu +{LB}_{j}),\;otherwise \end{array}\right.$$ (2)

$$MOP(C_{i})=1-\frac{{C_{i}}^{\frac{1}{\alpha }}}{{M_{iter}}^{\frac{1}{\alpha }}}$$ (3)

where *X*_*new*_(*i*,*j*) is the new solution to be computed, *best*(*j*) is the best solution from the previous iteration, *ϵ* is a very small integer, *UB*_*j*_ *and LB*_*j*_ are the upper and lower bound respectively. *μ* = 0.5 and *α* = 5 [11] are, respectively, the stochastic scaling factor and the exploitation accuracy over the iterations.

### 3.4. Exploitation phase

If *b*1>*MOA*, the exploitation phase is activated, executing either the ’+’ or the ’-’ operator. The candidate solutions are updated using these two operators, which are modeled in Eq 4. The high density of values generated by the operators makes them ideal for exploitation. The low dispersion values can search the neighborhood of already visited regions in the search space for optimal solutions. They are able to converge to the optimal solution, unlike the *’ln’* and *’e’* operators. In essence, the ’+’ and ’-’ operators are complementary.

A third beta distributed random number (*b*3) is generated, if *b*3<0.5, and the subtraction operator is executed. While the subtraction operator is executing, the addition operator is ignored. If *b*3≥0.5, the addition operator is executed, while also ignoring the subtraction operator. We used a stochastic scaling coefficient (*μ*) to increase the diversity of the addition or the subtraction values so as to explore more diverse regions of the search space. This helps nAOA avoid getting stuck in the local optima. Fig 3 shows how the candidate solutions are updated using the simple arithmetic rule, as shown in Eq 4.


$$X_{new}(i,j)=\left\{ \begin{array}{c} best(j)-\left(MOP\right)\times (({UB}_{j}-{LB}_{j})\times \mu +{LB}_{j}),\;b3<0.5\\ best(j)+(MOP)\times (({UB}_{j}-{LB}_{j})\times \mu +{LB}_{j}),\;otherwise \end{array}\right.$$ (4)

### 3.5. Pseudocode and computational complexity of nAOA

**Fig 4:**
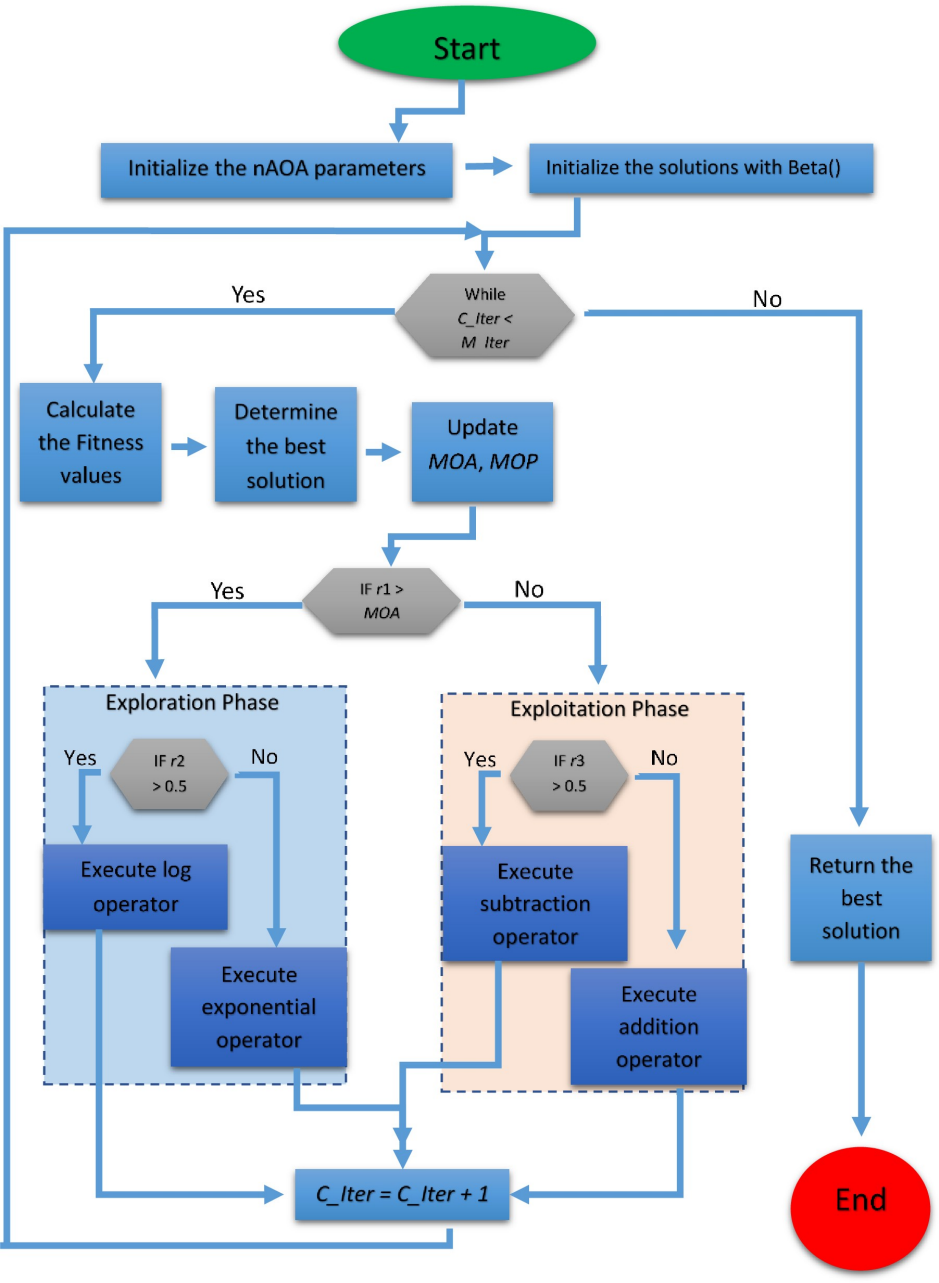
The nAOA flowchart.

In this section, we summarize the proposed improved arithmetic operator algorithm. The optimization process randomly executes the natural log (*ln*), exponential (*e*), addition (+), and subtraction (-) operators. The value of MOA is set between 0.2 to 0.9, which determines which phase the algorithm goes into. The algorithm avoids converging towards the near-optimal solution whenever *r*1>*MOA* and eventually stops after reaching a certain criterion as shown in the pseudocode below. Algorithm 1 shows the steps of our proposed algorithm, and the flow chart is given in Fig 4.

The main optimization processes of the algorithm are the initialization processes, evaluation of fitness function, and updating candidate solutions. The population size is *N*; updating the candidate solution depends on the iterations (*I*) and the different optimization problem parameters (*P*). Therefore, the computational complexity of nAOA is *O*(*N* × (*IP* + 1)).

**Algorithm 1**. Pseudocode of the nAOA

Set the values for *α*,*μ*.

*Use beta distribution to initialize the candidate solutions’ positions*. (*i* = 1,…,*N*.)

*Calculate the Fitness of each given solutions*

*Determined best solution so far*

***while*** (*t* < *Maximum Iteration*) do

    *Compute the MOA and MOP*

    ***for*** (*i* = 1 *to number of Solutions*) do

        ***for*** (*j* = 1 *to size of problem dimension*) do

        Generate *b*_1_,*b*_2_,*b*_3_ (beta distributed random values between [0,1]

***Exploration phase*:        *if****b*_1_ > *MOA*

                            ***if*** *b*_2_ > 0.5 then

                        Update the *i*^*th*^ solutions’ positions using the ***log*** *operator (**Eq* 2*)*.

                ***else***

                                Update the *i*^*th*^ solutions’ positions using the ***exp*** *operator (**Eq* 2*)*.

                ***end if***

                        ***else***

***Exploitation phase*:                *if b***_**3**_**> 0.5** then

                                    Update the *i*^*th*^ solutions’ positions using the (*S*"+") in Eq (*4*).

                        ***else***

                                 Update the *i*^*th*^ solutions’ positions using the (*A*"+") in Eq (*4*).

                            ***end if***

                        ***end if***

                ***end for***

            ***end for***

*Calculate the Fitness of each given solutions*

*Determined best so far*

*t* = *t*+1

***end while***

Return the best solution (*x*).

## 4. Results and discussion

In this section, we present the results of experiments conducted to evaluate the performance of nAOA. We used 20 benchmark test functions, 10 CEC 2020 test functions, and 3 engineering problems to evaluate the nAOA. We compared the results of nAOA on the 20 benchmark test functions, 10 CEC 2020 test functions, and engineering problems with the results from the original AOA and the following algorithms:

- Constriction coefficient-based PSO and GSA (CPSOGSA) [22]
- Gravitational search algorithm (GSA) [7]
- Particle swarm optimization (PSO) [9]
- Biogeography-based optimization (BBO) [23]
- Differential evolution (DE) [24]
- Ant colony optimization (ACO) [10]
- Salp swarm algorithm (SSA) [25]
- Sine cosine algorithm (SCA) [12]
- Grey wolf optimizer (GWO) [26]

The algorithms and engineering design problems were implemented using MATLAB R2020b; they were run on Windows 10 OS, Intel Core i7-7700@3.60GHz CPU, 16G RAM. The number of function evaluations was set at 50,000, and the number of independent runs was set at 30. The source codes are publicly available from the respective references. For a fair comparison, all the algorithms were executed using 1000 iterations and a population size of 50. The controlling parameters of the algorithms considered are given in Table 1. The test functions used for our experiments are presented in Tables 2 and 3. The results are presented using five (5) performance indicators: best, worst, average, standard deviation (SD), and median. The algorithms are compared using mean, standard deviation, Friedman ranking (Rank) test, and Wilcoxon signed-rank test.

**Table 1:** Controlling parameters for algorithms considered.

Algorithm	Name of the parameter	Value of the parameter
GSA	Elitist check (number of fittest agents after stopping criterion)	1
	Rpower (exponent of distance between agents)	1
	Min_flag (1: minimum; 0: maximum)	1
ACO	Pheromone update constant	1
	Initial pheromone	10
	Pheromone sensitivity	0.3
	Visibility sensitivity	0.1
AOA	*α*	5
	*μ*	0.05
DE	Lower bound of scaling factor	0.2
	Upper bound of scaling factor	0.8
	PCR (crossover probability)	0.8
PSO	C_1_, C_2_ (personal and social constants)	2
	Wmax (maximum inertia weight)	0.9
	Wmin (minimum inertia weight)	0.2
CPSOGSA	*<p1*, *<f>2* (control parameters)	2.05
BBO	nKeep (number of habitats retained after every generation)	0.2
	Pmutation (mutation probability)	0.9
GWO	a (area vector)	[0,2]
	r_1f_ r_2_ (random vectors)	[0,1]
SCA	a (constant)	2
SSA	c_2_, c_3_ (random numbers)	[0,1]

**Table 2:** Classical test functions.

ID	Type	Function	Dimension	Bounds	Global
F1	Unimodal	$f(x)=\sum_{i=1}^{n}x_{i}^{2}$	30	[–100,100]	0
F2	Unimodal	$f(x)=\sum_{i=0}^{n}|x_{i}|+\prod_{i=0}^{n}\left|x_{i}\right|$	30	[–10,10]	0
F3	Unimodal	$f(x)=\sum_{i=1}^{d}{\left(\sum_{j=1}^{i}x_{j}\right)}^{2}$	30	[–100,100]	0
F4	Unimodal	$f(x)={max}_{i}\{|x_{i}|,1\leq i\leq n\}$	30	[–100,100]	0
F5	Unimodal	$f(x)=\sum_{i=1}^{n-1}{\left[100(x_{i}-x_{i+1}\right)}^{2}+{\left(1-x_{i}\right)}^{2}]$	30	[–30,30]	0
F6	Unimodal	$f(x)=\sum_{i=1}^{n}{\left([x_{i}-0.5]\right)}^{2}$	30	[–100,100]	0
F7	Unimodal	$f(x)=\sum_{i=0}^{n}ix_{1}^{4}+rand[0,1)$	30	[–128,128]	0
F8	Multimodal	$f(x)=\sum_{i=1}^{n}-x_{i}sin(\sqrt{\left|x_{i}\right|})$	30	[–500,500]	-418.9829×*n*
F9	Multimodal	$f(x)=10+\sum_{i=1}^{n}{(x}_{i}^{2}-10cos(2\pi x_{i}))$	30	[-5.12,5.12]	0
F10	Multimodal	$f(x)=-a\;exp\left(-0.02\sqrt{n^{-1}\sum_{i=1}^{n}x_{i}^{2}}\right)-exp\left(n^{-1}\sum_{i=1}^{n}\cos\left(2\pi x_{i}\right)\right)+a+e,a=20$	30	[–32,32]	0
F11	Multimodal	$f(X)=1+\frac{1}{4000}\sum_{i=1}^{n}x_{i}^{2}-\prod_{i=1}^{n}cos(\frac{x_{i}}{\sqrt{i}})$	30	[–600,600]	0
F12	Multimodal	$f(x)=\frac{\pi }{n}\{10\sin\left(\pi y_{i}\right)\}+\sum_{i=1}^{n-1}{\left(y_{i}-1\right)}^{2}[1+10{sin}^{2}(\pi y_{i+1})+\sum_{i=1}^{n}u(x_{i},10,100,4)]$ Where $y_{i}=1+\frac{x_{i}+1}{4},u(x_{i},a,k,m)\left\{ \begin{array}{l} K{\left(x_{i}-a\right)}^{m}\;if\;x_{i}>a\\ 0\;-a\leq x_{i}\geq a\\ K{\left(-x_{i}-a\right)}^{m}-a\leq x_{i} \end{array}\right.$	30	[–50,50]	0
F13	Multimodal	$f(x)=0.1(\sin^{2}\left(3\pi x_{1}\right)+\sum_{i=1}^{n}{\left(x_{i}-1\right)}^{2}[1+{sin}^{2}(3\pi x_{i}+1)]+{{\left(x_{n}-1\right)}^{2}1+sin}^{2}(2\pi x_{n}))+\sum_{i=1}^{n}u(x_{i},5,100,4)$	30	[–50,50]	0
F14	Fixed-dimension multimodal	$f(x)={\left(\frac{1}{500}+\sum_{j=1}^{25}\frac{1}{j+\sum_{i=1}^{2}}(x_{i}-a_{ij})\right)}^{-1}$	2	[–65,65]	1
F15	Fixed-dimension multimodal	$f(x)=\sum_{i=1}^{11}{\left[a_{i}-\frac{x_{1}(b_{i}^{2}+b_{i}x_{2})}{b_{i}^{2}+b_{i}x_{3}+x_{4}}\right]}^{2}$	4	[–5,5]	0.00030
F16	Fixed-dimension multimodal	$f(x)=ax_{1}^{2}-2.1x_{1}^{4}+\frac{1}{3}x_{1}^{6}+x_{1}x_{2}-4x_{2}^{2}+4x_{2}^{4}$	2	[–5,5]	-1.0316
F17	Fixed-dimension multimodal	$f(x)={\left(x_{2}-\frac{5.1}{{4\pi }^{2}}x_{1}^{2}+\frac{5}{\pi }x_{1}-6\right)}^{2}+10(1-\frac{1}{8\pi })cosx_{1}+10$	2	[–5,5]	0.398
F18	Fixed-dimension multimodal	$f(X)=[1+{\left(x_{1}+x_{2}+1\right)}^{2}(19-14x_{1}+3{x_{1}}^{2}-14x_{2}+6x_{1}x_{2}+3{x_{2}}^{2})]\times [30+{\left(2x_{1}-3x_{2}\right)}^{2}(18-32x_{1}+12{x_{1}}^{2}+48x_{2}-36x_{1}x_{2}+27{x_{2}}^{2}]$	2	[–2,2]	3
F19	Fixed-dimension multimodal	$f(x)=-\sum_{i=1}^{4}c_{i}exp(-\sum_{i=1}^{3}a_{ij}{\left(x_{j}-p_{ij}\right)}^{2})$	3	[–1,2]	-3.86
F20	Fixed-dimension multimodal	$f(x)=-\sum_{i=1}^{4}c_{i}exp(-\sum_{i=1}^{6}a_{ij}{\left(x_{j}-p_{ij}\right)}^{2})$	6	[0,1]	-3.2

**Table 3:** Summary of the CEC2020 test suite.

Type	Number	Functions	$F_{i}^{*}$
Unimodal Function	F21	Shifted and rotated bent cigar function (CEC 2017^[^^4^^]^ F1)	100
Basic Functions	F22	Shifted and rotated Schwefel’s function (CEC 2014^[^^3^^]^ F11)	1100
	F23	Shifted and rotated Lunacek bi-Rastrigin function (CEC 2017^[^^4^^]^ F7)	700
	F24	Expanded Rosenbrock’s plus Griewangk’s function (CEC2017^[^^4^^]^f19)	1900
Hybrid Functions	F25	Hybrid function 1 (N = 3) (CEC 2014^[^^3^^]^ F17)	1700
	F26	Hybrid function 2 (N = 4) (CEC 2017^[^^4^^]^ F16)	1600
	F27	Hybrid function 3 (N = 5) (CEC 2014^[^^3^^]^ F21)	2100
CompositionFunctions	F28	Composition function 1 (N = 3) (CEC 2017^[^^4^^]^ F22)	2200
	F29	Composition function 2 (N = 4) (CEC 2017^[^^4^^]^ F24)	2400
	F30	Composition function 3 (N = 5) (CEC 2017^[^^4^^]^ F25)	2500

* Search range: [–100,100] ^D^.

### 4.1. Results for benchmark functions

The numerical efficiency of the proposed nAOA algorithm was tested by solving 30 mathematical optimization problems. The first 20 problems are classical benchmark functions, while the remaining 10 problems are composite benchmark functions from the CEC 2020 test suite, frequently used in the optimization literature. The benchmark functions can be divided into unimodal, multimodal, fixed-dimension multimodal, and composite functions. The major difference between fixed-dimensional multimodal functions and multimodal functions is their ability to tune the number of design variables. By contrast, the composite test functions make finding global optima challenging by shifting the global optimum to random positions. Tables 4 and 5 give the results for the benchmark and composite test functions, respectively.

**Table 4:** Classical benchmark functions.

Function	Value	CPSOGSA	GSA	PSO	BBO	DE	ACO	SSA	SCA	GWO	nAOA	AOA
**F1**	Best	3.07E-20	3.95E-19	2.18E-54	4.66E-08	2.77E-43	385	1.56E-10	2.15E-40	3.40E-149	0	0
	Worst	1.33E-19	2.48E-18	1.37E-46	2.72E-06	5.06E-41	385	1.01E-09	2.79E-29	3.80E-142	0	0
	Average	8.39E-20	1.30E-18	6.10E-48	5.01E-07	5.73E-42	385	5.61E-10	9.40E-31	3.84E-143	0	0
	SD	2.34E-20	5.34E-19	2.48E-47	5.50E-07	9.57E-42	0	2.09E-10	5.08E-30	9.78E-143	0	0
	Median	7.76E-20	1.15E-18	2.64E-49	3.45E-07	2.61E-42	385	5.69E-10	1.67E-34	3.14E-145	0	0
**F2**	Best	4.23E-10	2.36E-09	2.70E-29	3.20E-05	2.58E-25	3.63E+06	4.72E-06	2.49E-24	1.28E-83	0	0
	Worst	7.79E-10	5.30E-09	8.37E-26	0.000139	3.00E-24	3.63E+06	0.001054	2.05E-19	1.05E-79	0	0
	Average	6.36E-10	3.59E-09	4.20E-27	7.18E-05	1.36E-24	3.63E+06	4.22E-05	1.60E-20	9.27E-81	0	0
	SD	8.50E-11	7.93E-10	1.56E-26	2.96E-05	7.64E-25	0	0.000191	4.88E-20	2.28E-80	0	0
	Median	6.30E-10	3.51E-09	5.63E-28	6.29E-05	1.23E-24	3.63E+06	6.70E-06	2.66E-22	9.53E-82	0	0
**F3**	Best	8.60E-20	1.76E-18	9.91E-17	0.001513	0.028491	7949	3.81E-10	2.05E-21	4.51E-75	0	0
	Worst	4.91E-19	0.001491	8.97E-13	0.02492	0.68113	8097	2.61E-09	7.51E-13	3.96E-66	0	0
	Average	1.85E-19	4.97E-05	9.20E-14	0.007328	0.20667	8007.7	1.24E-09	5.51E-14	2.86E-67	0	0
	SD	8.25E-20	0.000272	2.11E-13	0.005159	0.14399	33.618	5.85E-10	1.56E-13	8.87E-67	0	0
	Median	1.74E-19	3.57E-18	8.91E-15	0.005741	0.18277	8002	1.16E-09	2.14E-16	3.56E-71	0	0
**F4**	Best	1.10E-10	4.93E-10	8.30E-15	0.002213	7.12E-08	10	8.07E-06	1.99E-13	1.10E-48	0	0
	Worst	2.18E-10	1.31E-09	4.30E-11	0.015541	3.78E-07	10	2.14E-05	2.38E-08	1.24E-44	0	0
	Average	1.69E-10	8.79E-10	3.69E-12	0.008172	1.68E-07	10	1.43E-05	2.26E-09	1.21E-45	0	0
	SD	3.11E-11	1.76E-10	8.60E-12	0.003127	6.67E-08	0	3.29E-06	5.70E-09	2.75E-45	0	0
	Median	1.74E-10	8.68E-10	1.17E-12	0.008299	1.53E-07	10	1.38E-05	2.69E-10	9.14E-47	0	0
**F5**	Best	1.2284	5.0105	0.055761	0.056942	0.068386	1.11E+06	2.029	6.0283	5.1566	1.2284	4.4762
	Worst	255.85	88.952	7.9865	14.874	6.6217	1.13E+06	244.94	8.0648	8.9099	5.1284	5.6396
	Average	36.361	8.2091	3.3821	4.6772	1.8597	1.12E+06	21.494	6.8968	6.4561	4.6145	5.1135
	SD	66.123	15.251	1.5961	4.0116	1.7585	5048.6	59.099	0.4814	0.69926	0.25943	0.28703
	Median	3.4962	5.4142	3.8446	3.8241	1.2368	1.12E+06	6.3983	6.8113	6.224	4.6371	5.1389
**F6**	Best	2.23E-20	8.11E-19	0	3.02E-08	0	442.5	1.80E-10	0.098421	2.03E-07	2.23E-20	0.003032
	Worst	1.46E-19	2.89E-18	0	2.44E-06	0	442.5	1.01E-09	0.54801	1.50E-06	0.003223	0.02523
	Average	8.47E-20	1.72E-18	0	5.75E-07	0	442.5	5.85E-10	0.27283	6.54E-07	0.001717	0.012978
	SD	2.96E-20	5.50E-19	0	5.72E-07	0	0	1.85E-10	0.1151	2.66E-07	0.000706	0.005789
	Median	8.25E-20	1.78E-18	0	3.99E-07	0	442.5	5.83E-10	0.23292	5.80E-07	0.001759	0.012989
**F7**	Best	0.000495	0.001583	0.000315	0.000239	0.000754	57853	0.000461	3.23E-05	2.76E-05	0.000495	8.47E-07
	Worst	0.012861	0.009237	0.002674	0.002435	0.004141	59858	0.011808	0.002274	0.0004708	0.000141	0.00013
	Average	0.005171	0.004395	0.000968	0.000726	0.002438	58753	0.003754	0.000773	0.0001735	4.86E-05	2.30E-05
	SD	0.003135	0.001793	0.000551	0.000478	0.00093	623.18	0.002704	0.000665	0.0001022	4.12E-05	2.59E-05
	Median	0.004614	0.004343	0.000904	0.000578	0.002275	58815	0.003585	0.000544	0.0001648	4.25E-05	1.65E-05
**F8**	Best	-3596.1	-2470.4	-3597.6	-3953	-4189.8	-24.036	-3479.2	-2619.9	-3527.1	-3596.1	-3850.5
	Worst	-2273.4	-1099.7	-2709.3	-3202.8	-4189.8	-24.036	-2463.4	-2021.6	-2348.8	-3608.4	-2866.2
	Average	-2985.5	-1570.5	-3102.4	-3596.3	-4189.8	-24.036	-2986.5	-2340.9	-2906	-3933.8	-3417.4
	SD	328.71	327.61	195.88	206.06	2.78E-12	1.08E-14	274.03	152.5	359.28	170.26	264.6
	Median	-3003.1	-1513.7	-3122.4	-3617.4	-4189.8	-24.036	-3054.8	-2356.7	-2797.8	-3978.4	-3400.2
**F9**	Best	8.9546	0.99496	3.9798	3.9798	0	385	4.9748	0	0	8.9546	0
	Worst	49.748	5.9698	21.889	14.924	0	385	27.859	7.46E-08	0	0	0
	Average	19.866	3.1507	9.5848	7.8938	0	385	14.825	2.49E-09	0	0	0
	SD	9.484	1.3847	3.9495	3.0904	0	0	5.6607	1.36E-08	0	0	0
	Median	18.904	2.9849	9.4521	7.4622	0	385	14.427	0	0	0	0
**F10**	Best	2.00E-10	8.08E-10	4.44E-15	2.91E-05	4.44E-15	14.218	4.45E-06	8.88E-16	4.44E-15	2.00E-10	8.88E-16
	Worst	4.87E-10	3.10E-09	7.99E-15	0.000599	4.44E-15	14.218	2.5799	4.44E-15	7.99E-15	8.88E-16	8.88E-16
	Average	3.40E-10	1.89E-09	4.80E-15	0.000227	4.44E-15	14.218	0.45561	4.09E-15	4.56E-15	8.88E-16	8.88E-16
	SD	6.83E-11	4.97E-10	1.08E-15	0.000123	0	5.42E-15	0.81522	1.08E-15	6.49E-16	0	0
	Median	3.34E-10	1.86E-09	4.44E-15	0.000203	4.44E-15	14.218	1.05E-05	4.44E-15	4.44E-15	8.88E-16	8.88E-16
**F11**	Best	0.063978	0	0.024637	0.004278	0	0.43794	0.076242	0	0	0.063978	0
	Worst	0.64029	0.31458	0.13046	0.090928	0	0.67057	0.70665	0.35098	0.08922	0.007483	0
	Average	0.23818	0.030425	0.065862	0.044379	0	0.54745	0.23328	0.026039	0.01517	0.000249	0
	SD	0.14922	0.060876	0.027713	0.021973	0	0.056981	0.12736	0.084842	0.020004	0.001366	0
	Median	0.18445	0.013544	0.066433	0.041822	0	0.54653	0.20914	0	0.0090838	0	0
**F12**	Best	3.9329	2.12E-19	2.75E-06	0.015191	1.03E-05	2.18E+09	2.9526	3.1554	0.026973	3.9329	0.49795
	Worst	18.832	1.2577	1.1423	0.030959	8.53E-05	2.18E+09	11.717	8.09E+06	0.11951	0.31117	0.67319
	Average	7.9747	0.35845	0.1075	0.021195	2.70E-05	2.18E+09	7.2727	4.72E+05	0.068205	0.27296	0.59617
	SD	3.5208	0.31492	0.22475	0.003743	1.75E-05	18.936	2.1124	1.50E+06	0.024278	0.023426	0.042977
	Median	7.0873	0.30048	0.062208	0.020807	2.00E-05	2.18E+09	7.4269	4871.2	0.064053	0.27241	0.6007
**F13**	Best	1.02E-20	8.17E-20	1.35E-32	2.05E-09	1.35E-32	97929	1.14E-11	0.079397	3.99E-07	1.02E-20	0.46953
	Worst	2.91E-20	3.26E-19	1.35E-32	3.66E-07	1.35E-32	97929	0.010987	0.35204	0.10037	0.76797	0.9949
	Average	1.80E-20	1.75E-19	1.35E-32	4.22E-08	1.35E-32	97929	0.002198	0.22937	0.009578	0.26741	0.77032
	SD	5.36E-21	7.10E-20	5.57E-48	6.69E-08	5.57E-48	0	0.00447	0.070419	0.029296	0.20027	0.16483
	Median	1.71E-20	1.62E-19	1.35E-32	2.33E-08	1.35E-32	97929	3.37E-11	0.23362	1.01E-06	0.23147	0.75585
**F14**	Best	-3596.1	-2470.4	-3597.6	-3953	-4189.8	-24.036	-3479.2	-2619.9	-3527.1	-3596.1	-3850.5
	Worst	-2273.4	-1099.7	-2709.3	-3202.8	-4189.8	-24.036	-2463.4	-2021.6	-2348.8	-3608.4	-2866.2
	Average	-2985.5	-1570.5	-3102.4	-3596.3	-4189.8	-24.036	-2986.5	-2340.9	-2906	-3933.8	-3417.4
	SD	328.71	327.61	195.88	206.06	2.78E-12	1.08E-14	274.03	152.5	359.28	170.26	264.6
	Median	-3003.1	-1513.7	-3122.4	-3617.4	-4189.8	-24.036	-3054.8	-2356.7	-2797.8	-3978.4	-3400.2
**F15**	Best	0.000307	0.001277	0.000307	0.000308	0.000386	0.40836	0.00044	0.000337	0.0003075	0.000307	0.000346
	Worst	0.020363	0.010464	0.020363	0.001355	0.000755	0.40836	0.001241	0.001429	0.020363	0.073329	0.02603
	Average	0.003334	0.00397	0.00476	0.000632	0.000643	0.40836	0.000879	0.000914	0.0043187	0.009563	0.004905
	SD	0.006795	0.002405	0.007946	0.000199	9.99E-05	1.13E-16	0.000282	0.000387	0.0081595	0.019043	0.008062
	Median	0.000743	0.003055	0.001223	0.000634	0.000645	0.40836	0.00076	0.000771	0.0003075	0.000579	0.000484
**F16**	Best	1.02E-20	8.17E-20	1.35E-32	2.05E-09	1.35E-32	97929	1.14E-11	0.079397	3.99E-07	1.02E-20	0.46953
	Worst	2.91E-20	3.26E-19	1.35E-32	3.66E-07	1.35E-32	97929	0.010987	0.35204	0.10037	0.76797	0.9949
	Average	1.80E-20	1.75E-19	1.35E-32	4.22E-08	1.35E-32	97929	0.002198	0.22937	0.009578	0.26741	0.77032
	SD	5.36E-21	7.10E-20	5.57E-48	6.69E-08	5.57E-48	0	0.00447	0.070419	0.029296	0.20027	0.16483
	Median	1.71E-20	1.62E-19	1.35E-32	2.33E-08	1.35E-32	97929	3.37E-11	0.23362	1.01E-06	0.23147	0.75585
**F17**	Best	0.000495	0.001583	0.000315	0.000239	0.000754	57853	0.000461	3.23E-05	2.76E-05	0.000495	8.47E-07
	Worst	0.012861	0.009237	0.002674	0.002435	0.004141	59858	0.011808	0.002274	0.0004708	0.000141	0.00013
	Average	0.005171	0.004395	0.000968	0.000726	0.002438	58753	0.003754	0.000773	0.0001735	4.86E-05	2.30E-05
	SD	0.003135	0.001793	0.000551	0.000478	0.00093	623.18	0.002704	0.000665	0.0001022	4.12E-05	2.59E-05
	Median	0.004614	0.004343	0.000904	0.000578	0.002275	58815	0.003585	0.000544	0.0001648	4.25E-05	1.65E-05
**F18**	Best	3	3	3	3	3	2275	3	3	3	3	3
	Worst	3	3.0274	3	30	3	2275	3	3.0001	3	84	30
	Average	3	3.0009	3	4.8	3	2275	3	3	3	12.9	9.2535
	SD	1.13E-15	0.004995	1.47E-15	6.8501	2.09E-15	0	7.31E-14	1.17E-05	2.96E-06	21.835	11.532
	Median	3	3	3	3	3	2275	3	3	3	3	3
**F19**	Best	2.00E-10	8.08E-10	4.44E-15	2.91E-05	4.44E-15	14.218	4.45E-06	8.88E-16	4.44E-15	2.00E-10	8.88E-16
	Worst	4.87E-10	3.10E-09	7.99E-15	0.000599	4.44E-15	14.218	2.5799	4.44E-15	7.99E-15	8.88E-16	8.88E-16
	Average	3.40E-10	1.89E-09	4.80E-15	0.000227	4.44E-15	14.218	0.45561	4.09E-15	4.56E-15	8.88E-16	8.88E-16
	SD	6.83E-11	4.97E-10	1.08E-15	0.000123	0	5.42E-15	0.81522	1.08E-15	6.49E-16	0	0
	Median	3.34E-10	1.86E-09	4.44E-15	0.000203	4.44E-15	14.218	1.05E-05	4.44E-15	4.44E-15	8.88E-16	8.88E-16
**F20**	Best	2.23E-20	8.11E-19	0	3.02E-08	0	442.5	1.80E-10	0.098421	2.03E-07	2.23E-20	0.003032
	Worst	1.46E-19	2.89E-18	0	2.44E-06	0	442.5	1.01E-09	0.54801	1.50E-06	0.003223	0.02523
	Average	8.47E-20	1.72E-18	0	5.75E-07	0	442.5	5.85E-10	0.27283	6.54E-07	0.001717	0.012978
	SD	2.96E-20	5.50E-19	0	5.72E-07	0	0	1.85E-10	0.1151	2.66E-07	0.000706	0.005789
	Median	8.25E-20	1.78E-18	0	3.99E-07	0	442.5	5.83E-10	0.23292	5.80E-07	0.001759	0.012989
p-value (0.000)	Friedman mean rank	6.53	6.95	4.58	6.1	4.22	11	7.28	6.43	4.9	3.48	4.55
	General mean rank	8	9	4	6	2	11	10	7	5	1	3

**Table 5:** Composite functions CEC 2020.

Function	Value	CPSOGSA	GSA	PSO	BBO	DE	ACO	SSA	SCA	GWO	nAOA	AOA
**F21**	Best	402.82	402.82	127.78	166.61	187.55	2.57E+10	110.88	3.37E+08	17636	100.14	4515.7
	Worst	1.14E+09	10757	8865.3	6807.1	11957	2.58E+10	10897	2.26E+09	3.94E+08	2453.8	16717
	Average	6.03E+07	7362.8	2693.4	1809	3092.9	2.58E+10	3546.4	9.25E+08	5.06E+07	539.16	7871.3
	SD	2.53E+08	1802.8	2591.3	1787.1	3089.6	3.51E+07	3130.4	3.96E+08	1.20E+08	679.2	3089.7
	Median	4820.3	7329.4	1584.6	1388.5	1909.6	2.58E+10	2584.2	8.53E+08	2.29E+05	310.32	7387.3
**F22**	Best	1708.3	1868.6	1140	1526.7	1361.5	3381.4	1591.5	2099.5	1239.2	1708.3	1341
	Worst	3018.4	3244.1	1944.6	2694.1	1849.6	3579	2554.7	2858.3	2199.1	2265.3	2103.2
	Average	2338.9	2331.9	1511.8	1897.3	1611.5	3475	2043.6	2446.2	1623.4	1852.9	1752.4
	SD	381.52	335.1	205.63	306.62	136.62	58.291	303.72	188.77	265.72	252.97	193.19
	Median	2338.4	2275.8	1474.2	1859.3	1602.4	3473	1928.9	2427.8	1628	1876.3	1709.5
**F23**	Best	747.51	712.78	713.81	716.63	718.45	816.31	718.4	762.05	715.27	747.51	761.19
	Worst	907.69	735.69	733.18	748.02	730.52	835.57	769.78	796.06	760.41	834.84	813.97
	Average	806.25	718.88	721.8	733.93	724.86	830	739.21	779.2	735.1	758.37	787.03
	SD	41.133	5.4895	5.7184	8.9233	3.7642	4.1447	13.567	9.969	13.226	22.909	15.727
	Median	792.59	717.26	720.8	732.52	725.34	830.38	737.89	778.89	736.24	756.92	791.24
**F24**	Best	1900.1	1900	1900.5	1900.3	1900.8	7.32E+05	1900.7	1907.2	1900.7	1900.1	1926.8
	Worst	2254.3	1902.8	1902.5	1907.2	1902.3	7.43E+05	1904	2186.7	1991.1	1996.9	2008.9
	Average	1935.6	1900.9	1901.4	1903	1901.7	7.37E+05	1901.7	1945.6	1907	1966.2	1960.2
	SD	89.268	0.69602	0.63295	1.5195	0.36594	2895.3	0.81329	58.785	19.848	18.886	18.957
	Median	1902.2	1900.7	1901.3	1903	1901.7	7.36E+05	1901.6	1931.7	1902.8	1964.9	1958.1
**F25**	Best	2409.8	1.84E+05	2334	3018.4	6937.4	2.34E+07	2787.2	13945	2983.8	2409.8	3901.9
	Worst	3.77E+05	8.67E+05	12394	1.07E+06	2.03E+05	2.34E+07	31779	3.09E+05	6.12E+05	46382	22483
	Average	99683	4.76E+05	5441.5	2.49E+05	54243	2.34E+07	7819.4	70448	1.37E+05	10929	10151
	SD	1.25E+05	2.01E+05	2759.6	3.15E+05	53909	95.544	6853	71390	2.14E+05	9217	4529.9
	Median	51320	4.56E+05	4730.2	44649	38270	2.34E+07	5275.5	39653	7666.8	8355.8	8534.4
**F26**	Best	1600.5	1611	1600	1600.6	1600	1861.5	1600	1600.8	1600.3	1600.5	1600.3
	Worst	1980.8	1744	1659.5	1661	1601.3	1862	1602.8	1602.8	1658.5	1618.8	1617.8
	Average	1667.2	1659.1	1625.7	1618.4	1600.4	1861.7	1601	1601.6	1606.9	1604.9	1604.6
	SD	95.234	32.899	23.51	22.832	0.37058	0.25407	0.84072	0.57677	13.972	7.4591	6.9393
	Median	1618.7	1659.3	1617.3	1610.8	1600.2	1861.5	1600.7	1601.5	1600.8	1601.1	1600.8
**F27**	Best	2546.9	12007	2429	3426.7	2281.3	1.39E+09	2331.8	4245.8	2975.4	2546.9	2349
	Worst	32441	1.64E+06	9453.7	2.43E+05	11028	1.39E+09	24342	36162	23259	34207	12858
	Average	10581	5.16E+05	4608.3	37405	5198.6	1.39E+09	7505.3	16337	11825	10471	5696.1
	SD	9700.1	4.40E+05	2222.4	54643	2780.1	13.869	6447.9	8265	6001.3	9669.5	2760.1
	Median	6283.6	4.32E+05	4156.7	20598	4349.5	1.39E+09	4252.2	15992	13426	7032.4	5658.8
**F28**	Best	2269.4	2300	2300.6	2301	2239.1	4551.9	2234	2307.1	2301.1	2269.4	2297.4
	Worst	4161.1	2300.8	3030.6	2758.3	2303.3	4622.7	2308.9	2571.1	2948.5	2540.9	2547
	Average	2593.2	2300.3	2367.4	2325.9	2297	4591	2296.8	2401.8	2356.8	2382	2412
	SD	644.17	0.23812	202.83	101.79	15.279	17.025	20.63	60.899	155.2	69.982	75.162
	Median	2306.2	2300.3	2301.9	2302.9	2301.5	4596.2	2302.6	2385	2306.6	2383	2408.2
**F29**	Best	2697.5	2500	2500	2500	2637.4	3337	2500	2563.2	2727.8	2697.5	2500.1
	Worst	2841.3	2876.6	2762.6	2791.3	2755	3343	2779.1	2815.8	2790.5	2840.6	2824.5
	Average	2786.4	2657.8	2712	2725.6	2737.2	3340	2739.3	2780.8	2751.2	2729.9	2696.8
	SD	35.984	154.81	79.413	85.55	33.535	1.6637	57.41	52.117	18.302	119.78	120.7
	Median	2790.9	2678.3	2742	2758.2	2749	3340.1	2751.8	2790.3	2744.8	2782.4	2760.3
**F30**	Best	2897.8	2899.6	2898.2	2897.7	2899	4650.7	2897.8	2940.7	2900.2	2897.8	2897.7
	Worst	3024.3	2943.6	2950	2953.5	2947.1	4670.9	2949.4	3016.1	2984.8	2996.8	2989.4
	Average	2939.4	2939.1	2925	2932.1	2926.1	4661.6	2917.9	2973.5	2934.4	2934.9	2927.4
	SD	37.551	13.494	24.26	22.653	19.621	4.8539	24.314	22.44	19.854	34.674	31.083
	Median	2945.5	2943.4	2944	2944.2	2930.2	4662.1	2899.4	2968.4	2941.1	2949.6	2913.4
p-value (0.000)	Friedman’s mean rank	8.7	5.2	3.15	5.5	3.85	11	3.85	8	6.2	2.9	6.1
	General mean rank	9	4	2	5	3	10	3	8	7	1	6

#### 4.1.1. Evaluation of exploitation capability

We discuss the exploitation ability of nAOA using unimodal functions F1–F7, since they have only one global optimum. It can be seen from Table 4 that nAOA outperformed the original AOA and nine other state-of-the-art algorithms considered for these functions. Though, all the algorithms were able to find the optimal solution as the best results, the performance, superiority, and stability of nAOA is confirmed by the value of the standard deviation, mean, and result of Friedman’s test. The mean values were used for the Friedman’s test, and a p-value of 0.00 was returned, which is less than the tolerance level of 0.05 hence, we reject the null hypothesis (the distributions of the obtained results for all the algorithms considered are the same). The nAOA returned the lowest mean rank, which means it performed optimally when compared with the ten other algorithms. This result also confirms nAOA’s ability to perform exploitation.

#### 4.1.2. Evaluation of exploration capability

The multimodal functions have many local optima and so provide a good test for the exploration capability of optimizers. Functions F8–F20 are multimodal and fixed-dimension multimodal functions. The number of local optima for each increase exponentially with the number of problem design variables. We can see from Table 4 that nAOA performed optimally and, in most cases, returned the lowest mean value and standard deviation. The stability of nAOA is also confirmed by the value of the standard deviation and result of Friedman’s test. The p-value of 0.00 was returned, which is less than the tolerance level of 0.05 hence, we reject the null hypothesis (the distributions of the obtained results for all the algorithms considered are the same). Again, nAOA returned the lowest mean rank of the Friedman’s test; indicating that it performed optimally when compared with 10 other algorithms. This also indicated that nAOA also has a good exploration capability.

#### 4.1.3. Ability to escape from local minima

We used the composite functions found in the CEC2020 suite to evaluate the ability of nAOA to escape local minima. A proper balance of exploration and exploitation guarantees avoidance of local optima. The results presented in Table 5 show that nAOA outperformed the original AOA, along with the nine algorithms considered for all the functions. It returned the lowest mean and standard deviation. The stability of nAOA is also confirmed by the value of the standard deviation and result of Friedman’s test. The p-value of 0.00 was returned, which is less than the tolerance level of 0.05 hence, we reject the null hypothesis (the distributions of the obtained results for all the algorithms considered are the same). Once more, the nAOA returned the lowest mean rank, which means it performed optimally when compared with 10 other algorithms. This proves that nAOA has a good balance of exploration and exploitation. This ability can be attributed to the update mechanism used by the proposed algorithm.

#### 4.1.4. Convergence behavior

**Fig 5:**
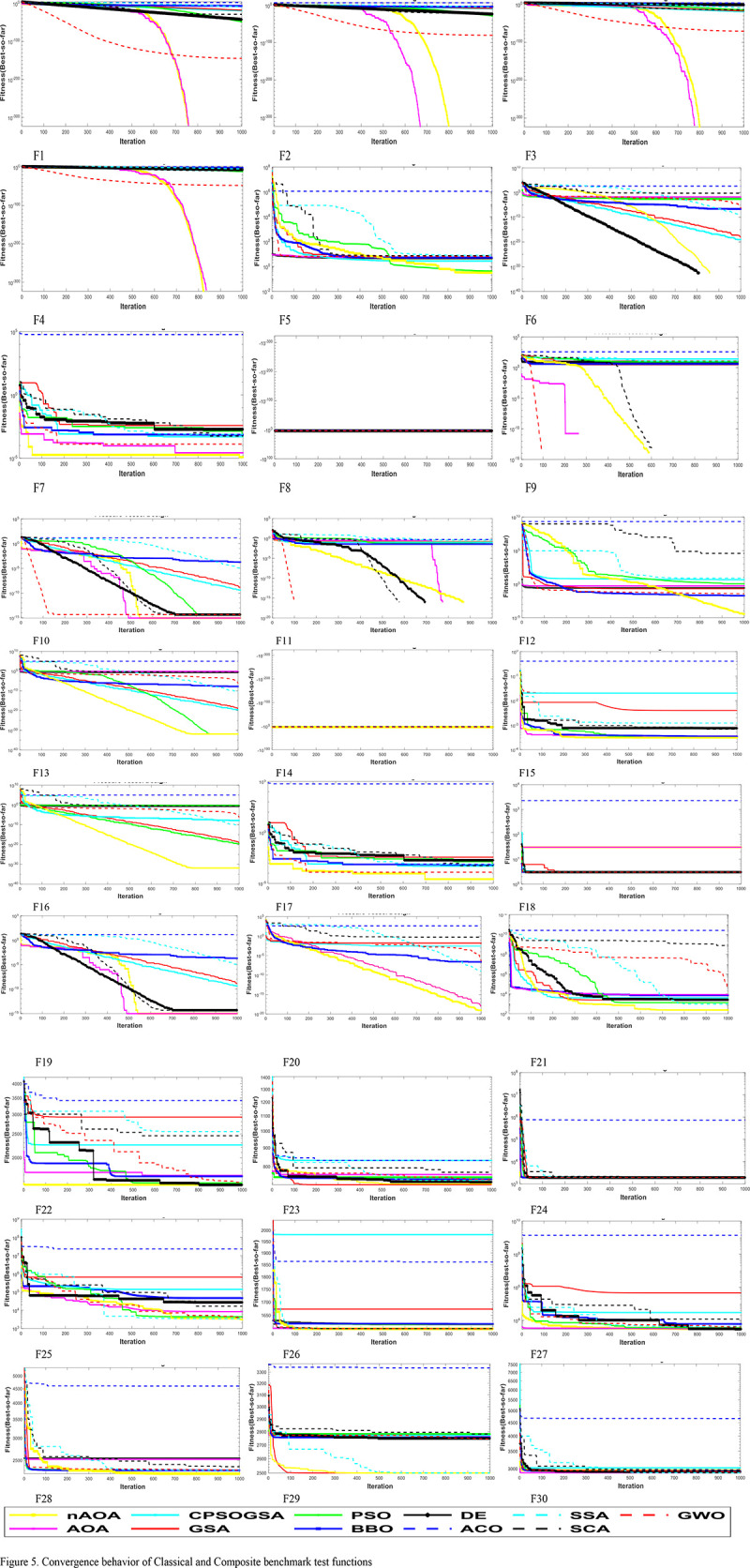
Convergence behavior of classical and composite benchmark test functions.

The convergence behavior of nAOA is compared with that of the original AOA and nine other state-of-the-art algorithms in Fig 5. It can be seen that nAOA tends to search extensively the areas with the likelihood of finding the global optima. For F1-F4, the algorithms did not converge abruptly to the earliest found best solutions. This behavior guarantees exploration and eventual convergence after multiple iterations. We can also see that nAOA converged to the optimal solution for these functions. The second behavior that can be noticed in the convergence is that, as iterations increase, the algorithms tend to be accelerated quickly towards the best solution found so far. The adaptive mechanism of the algorithms ensures they look for regions with a high likelihood of finding the optimal solution and, as such, converge more rapidly towards the optimum early in the iterations. This behavior is evident in F5, F12, F13, and F18. Another observed behavior is noticed in F16, F17, F19-F20, where the convergence occurs towards the final iterations. This can be attributed to the efforts of the algorithm to avoid local optima, so the search process continues till the end. The convergence curve for the composite function F21-F30 clearly confirms nAOA’s ability to escape the local minima. Accordingly, nAOA obtained superior and highly competitive results which are characterized by nAOA’s being able to converge towards the best result for all functions.

### 4.2. Application to engineering problem

Applying optimization techniques to engineering problems is primarily intended to minimize the values of design parameters and hence the overall design cost. The nAOA was applied to three mechanical engineering design problems: the welded beam design problem (WBD), the compression spring design problem (CSD), and the pressure vessel design problem (PVD). The penalty method has been adopted for constraint handling, whereby the algorithm is penalized for any constraint violation. Simple scalar penalty functions were adopted for this experiment.

The result obtained for the application of nAOA to solve the engineering problem was compared with 10 other metaheuristic algorithms: CPSOGSA, GSA, PSO, BBO, DE, ACO, GWO, SCA SSA, and AOA. For a fair comparison, the algorithms and engineering design were implemented in MATLAB R2020b; they were run on Windows 10 OS, Intel Core i7-7700@3.60GHz CPU, 16G RAM. The number of function evaluations was set at 50,000, and the number of independent runs was set at 30. The source codes are publicly available in the respective references. For a fair comparison, all the algorithms were executed using 1000 iterations and population size of 50. The results for each engineering problem are presented using five (5) performance indicators: namely, best, worst, average, standard deviation (SD), and median. In addition, the algorithms are compared using mean, standard deviation, and Wilcoxon signed-rank test.

#### 4.2.1. The welded beam design problem

**Fig 6:**
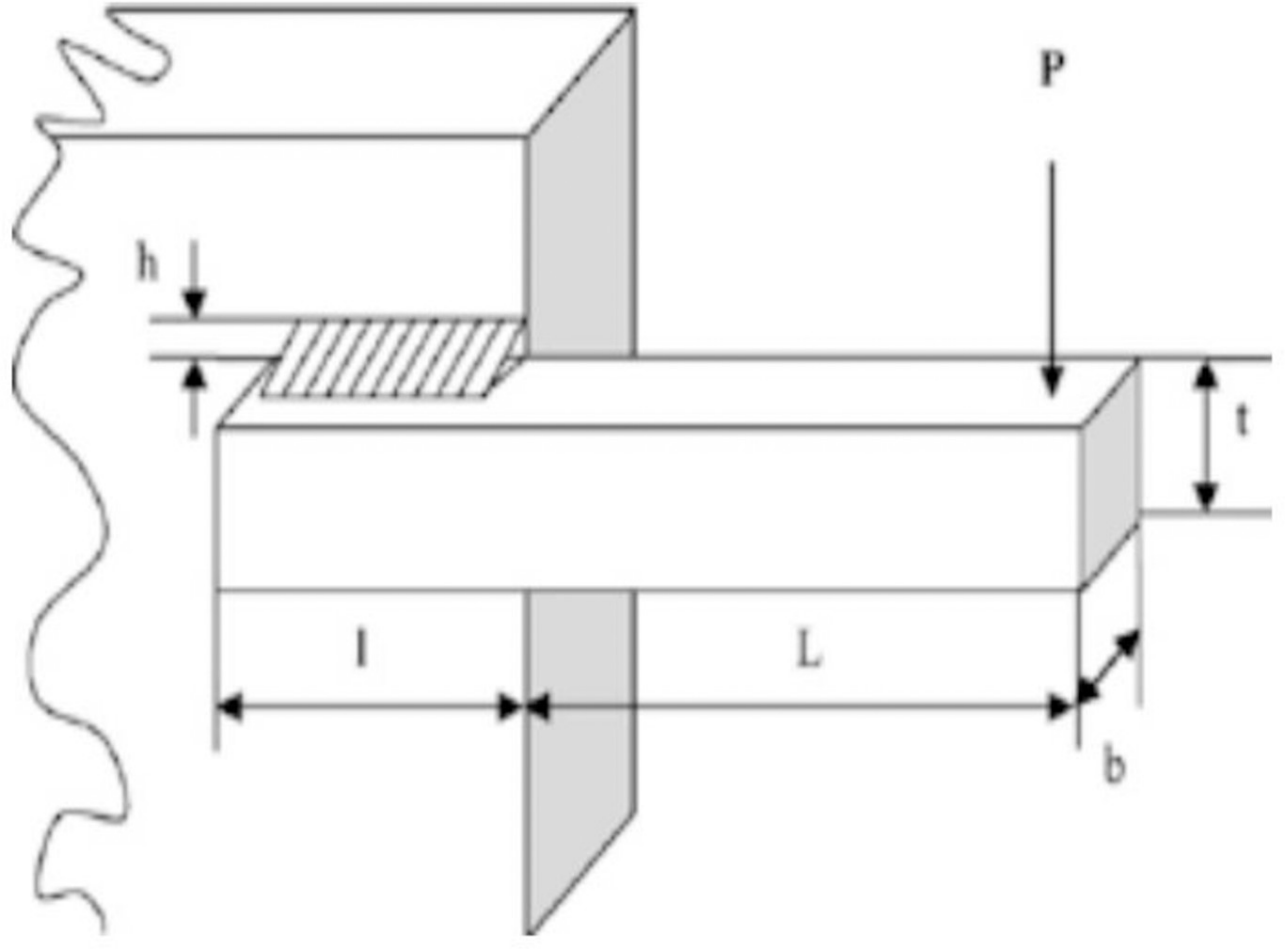
Design of WBD problem [28].

The welded beam design problem is a minimization problem, in which we used nAOA along with 10 other algorithms to reduce the manufacturing cost of the design [27]. Fig 6 gives an illustration of the WBD. The WBD constraints are shear (*τ*) and beam blending (*θ*) stress, bar buckling load (*P*_*c*_), beam end deflection (*δ*), and side constraints.

The design variables for WBD are:

$$\left[\begin{array}{c} x_{1}\\ x_{2}\\ x_{3}\\ x_{4} \end{array}\right]=\left[\begin{array}{c} h\\ l\\ t\\ b \end{array}\right]\;with\;the\;length\;(l),height\;(t),thickness\;(b)\;and\;weld\;thickness\;(h)\;of\;the\;bar..$$

The WBD problem is formulated mathematically as follows [27]:

$$Given\;\vec{l}=[l_{1}l_{2}l_{3}l_{4}]=[hltb]=[x_{1}x_{2}x_{3}x_{4}]$$

$$\min\;f(\vec{l})=l_{1}^{2}l_{2}1.10471+0.04811l_{3}l_{4}(14.0+l_{2})$$ (5)

subject to

$$s_{1}(\vec{l})=\tau (\vec{l})-\tau_{max}\leq 0,$$

$$s_{2}(\vec{l})=\sigma (\vec{l})-\sigma_{max}\leq 0,$$

$$s_{3}(\vec{l})=\delta (\vec{l})-\delta_{max}\leq 0,$$

$$s_{4}(\vec{l})=l_{1}-l_{4}\leq 0,$$

$$s_{5}(\vec{l})=P-P_{c}(\vec{l})\leq 0,$$

$$s_{6}(\vec{l})=0.125-l_{1}\leq 0,$$

$$s_{7}(\vec{l})=1.10471l_{1}^{2}+0.04811l_{3}l_{4}(14.0+l_{2})-5.0\leq 0.$$

The intervals for the design variables are as follows:

$$0.1\leq l_{1}\leq 2,$$

$$0.1\leq l_{2}\leq 10,$$

$$0.1\leq l_{3}\leq 10,$$

$$0.1\leq l_{4}\leq 2$$

where

$$\tau (\vec{l})=\sqrt{{\tau^{\prime }}^{2}+2\tau^{\prime }\tau^{\prime \prime (\frac{l_{2}}{R})}+{\left(\tau^{\prime \prime }\right)}^{2}}$$

$$\tau^{\prime }=P/\sqrt{2l_{1}l_{2}}$$

$$\tau^{\prime \prime }=MR/J$$

$$M=P\left(L+\frac{l_{2}}{2}\right)$$

$$J=2\left\{\sqrt{2}l_{1}l_{2}[(\frac{\left(l_{2}^{2}\right)}{4})+{\left(l_{1}+\frac{l_{3}}{2}\right)}^{2}]\right\}$$

$$P_{c}(\vec{l})=\frac{4.013E\sqrt{\frac{l_{3}^{2}l_{4}^{6}}{36}}}{L^{2}}\left(1-\frac{l_{3}}{2L}\sqrt{E}/4G\right).$$

The parameters for WBD are set as follows:

$$\sigma_{max}=3000psi,\;P=6000lb,\;L=14\;in,\;\delta_{max}=0.25in,\;E={3\times 10}^{6}psi,\;\tau_{max}=13600\;psi,\;and\;G=12\times 10^{6}psi.$$

The results of the experiment conducted for WBD for our comparative analysis are given in Table 6, which shows the results for nAOA and 10 other algorithms (GSA, PSO, BBO, DE, ACO, CPSOGSA, GWO, SCA, SSA, and AOA). The results indicate that nAOA outperformed the original AOA for the cost function of the WBD problem. Moreover, nAOA returned minimum values for mean and SD when compared with AOA. Looking at the values for the design variables *h*, *t*, and *b*, we see that nAOA returned optimal values for all three variables. However, the overall best performing algorithm in terms of the average and standard deviation is GWO. Nevertheless, our proposed algorithm was very competitive as it returned the same best cost value with GWO and came second to GWO for the mean and standard deviation. The Wilcoxon signed-rank test indicates that PSO, BBO, DE, ACO simulation results are not statistically significant because they have p-values greater than 0.05. Whereas those of SSA, SCA, GWO, nAOA, AOA, CPSOGSA, and GSA are significant because they have p-values less than 0.05.

**Table 6:** Results for experiment conducted for WBD.

	*H*	*l*	*t*	*b*	Best	Worst	Average	SD	Median	p-values
CPSOGSA	0.201423	3.121684	9.850499	0.201999	1.6976	2.2841	1.8545	0.13711	1.816	0.000
GSA	0.163725	9.282428	7.109815	0.352461	2.12	3.6595	2.8397	0.41809	2.8412	0.002
PSO	0.785289	1.997506	2	2	1.09E+14	1.09E+14	1.09E+14	0.047676	1.09E+14	0.10
BBO	0.959489	1.611894	2	2	1.09E+14	1.09E+14	1.09E+14	0.14365	1.09E+14	0.22
DE	0.784177	2	2	2	1.09E+14	1.09E+14	1.09E+14	0.047676	1.09E+14	0.31
ACO	1	4	3	2	1.69E+05	1.69E+05	1.69E+05	2.96E-11	1.69E+05	0.1
SSA	0.185461	3.654591	9.037761	0.205724	1.7084	2.6689	2.1938	0.22237	2.2059	0.001
SCA	0.191322	3.28746	10	0.204763	1.7366	1.8905	1.8197	0.036758	1.8198	0.000
GWO	0.20542	3.263227	9.036302	0.205746	**1.6957**	1.703	**1.6976**	**0.001619**	1.6972	0.000
nAOA	0.125228	10	10	0.204985	**1.6976**	1.9963	**1.7731**	**0.07421**	1.7433	0.000
AOA	0.204292	4.111883	10	0.20342	1.8603	2.8294	2.3488	0.26637	2.4734	0.001

**Fig 7:**
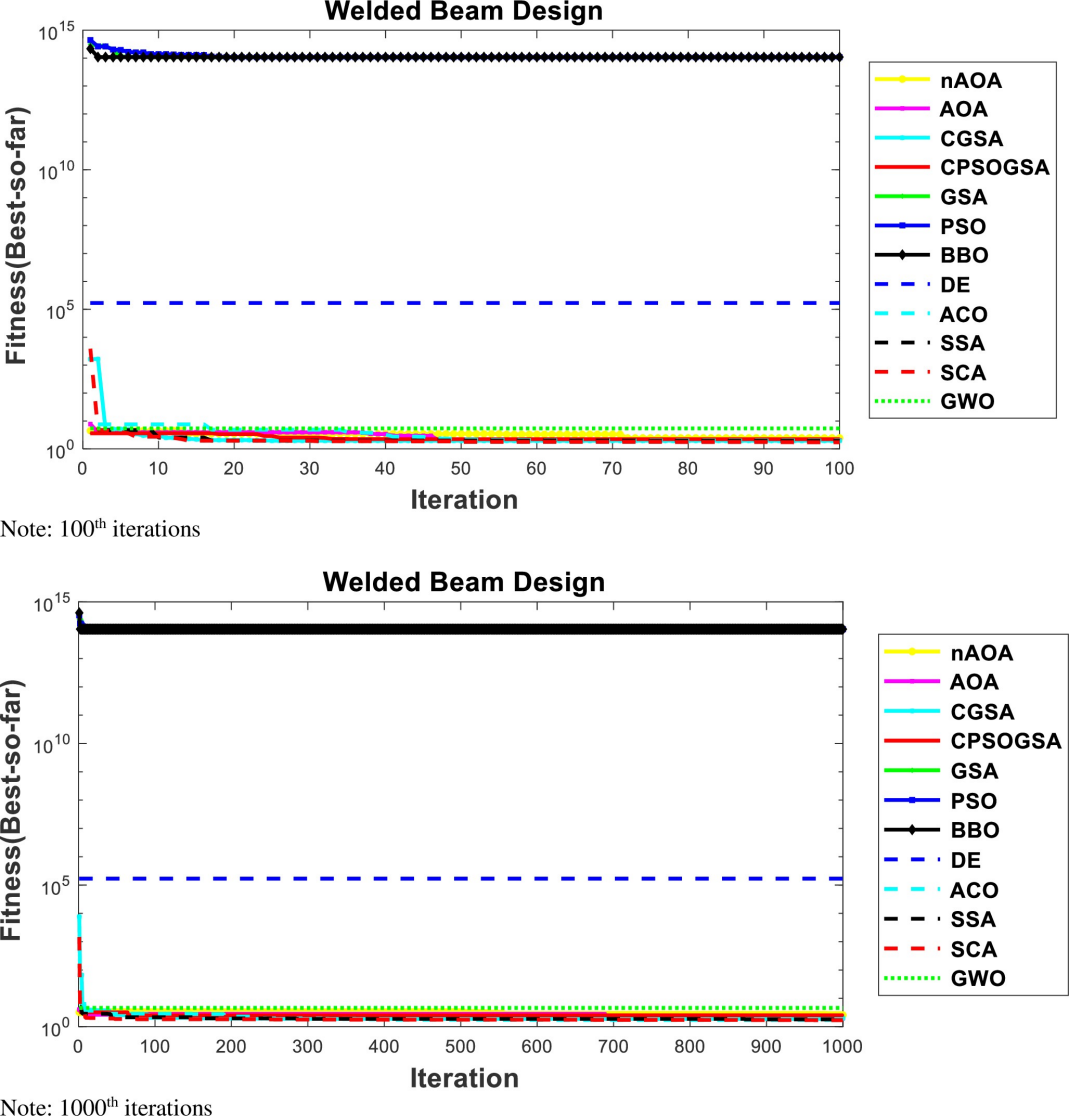
Convergence curves for WBD. Note: 100^th^ iterations. Note: 1000^th^ iterations.

The convergence curves at the 100^th^ and 1000^th^ iterations for nAOA and 10 other algorithms used for the comparative analysis is shown in Fig 7. We used these two curves to evaluate the algorithms’ behavior at both the early stage and later stage of the iterations. It shows that nAOA has regular values at the start of the iterations; the same can be observed for the other comparative algorithms. Since the algorithms were all able to find best results early in the iteration process, they converged towards the best result and remained stable until the end of the optimization iterations. The similar results at the different iteration phases show insensitivity to the initialization scheme used by the initial candidate solution. On one hand we notice that the convergence curves of nAOA, AOA, CGSA, GWO, SCA, SSA, and CPSOGSA lie close to each other as they have nearly equal values for the cost function. On the other hand, the convergence curve for PSO, BBO, and ACO lie together at the top of the figure because they all have large values for the average, SD, and median, which translate into sub-optimal results for the cost. We can see DE standing alone at the middle of the curves, where, although it returned a suboptimal result, it was still better than PSO, BBO, and ACO.

#### 4.2.2. Compression spring design problem

**Fig 8:**
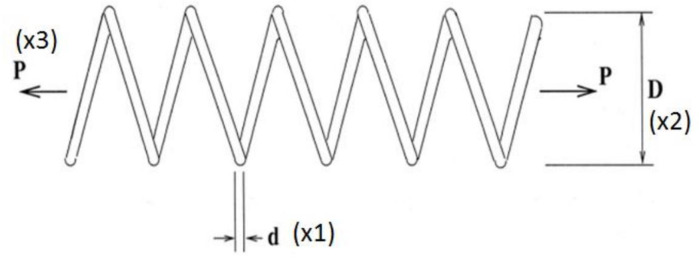
Compression spring design problem [27].

The compression spring design problem (CSD), as shown in Fig 8, is a continuous constrained optimization problem. The goal is to minimize the volume *V* of a coil spring under a constant tension/compression load. There are three design variables:

- the number of spring’s active coils *P* = *x*1 ∈ [2, 15]
- the diameter of the winding *D* = *x*2 ∈ [0.25, 1.3]
- the diameter of the wire *d* = o*x*3 ∈ [0.05, 2]

The mathematical formulation of the CSD problem is as given in [29].Given

$$l=[l_{1}l_{2}l_{3}]=[dDP]$$

$$Min\;f(\vec{l)}=(l_{3}+2)l_{2}l_{1}^{2}$$ (6)

subject to

$$s_{1}(\vec{l)}=1-\frac{l_{2}^{3}l_{3}}{717851^{4}}\leq 0,$$

$$s_{2}(\vec{l)}=\frac{4l_{2}^{2}-l_{1}l_{2}}{12566(l_{3}l_{1}^{3}-l_{1}^{4})}+1/{5108l_{1}}^{2}\leq 0,$$

$$s_{3}(\vec{l)}=1-\frac{140.45l_{1}}{l_{2}^{2}l_{3}}\leq 0,$$

$$s_{4}(\vec{l)}=\frac{l_{1}+l_{2}}{1.5}-1\leq 0.$$

The intervals for the design variables are:

$${0.05\leq l}_{1}\leq 2.00,$$

$$0.25\leq l_{2}\leq 1.30,$$

$$2.00\leq l_{3}\leq 15.0$$

The results used for our comparative analysis for CSD are shown in Table 7 for nAOA and 10 other algorithms (GSA, PSO, BBO, DE, ACO, CPSOGSA, GWO, SCA, SSA, and AOA). It can be seen that nAOA outperformed the original AOA for the cost function of the CSD problem. Moreover, nAOA returned smaller values for both mean and SD than did AOA. The same can be observed for the design variables *d*, *D*, and *P*. However, the overall best performing algorithm in terms of the best, average, and standard deviation is again the GWO. And once again, our proposed algorithm was very competitive in returning the same best cost value as did GWO and came second to GWO for mean and standard deviation. The PSO, BBO, ACO, and DE have large values for the average, SD, and median, translating to sub-optimal results for the cost. The Wilcoxon signed-rank test indicates that PSO, BBO, DE, ACO simulation results are not statistically significant because they have p-values greater than 0.05. The results of SSA, SCA, GWO, nAOA, AOA, CPSOGSA, and GSA were significant because they have p-values less than 0.05.

**Table 7:** Results for experiment conducted for CSD.

	*D*	*D*	*P*	Best	Worst	Average	SD	Median	p-values
CPSOGSA	0.138911	1.29213	12.02621	3.6619	3.7263	3.6668	0.015784	3.6619	0.001
GSA	0.124335	1.3	9.641125	3.7502	12.411	6.3644	2.4806	5.3726	0.021
PSO	2	2	2	409.77	409.77	409.77	2.89E-13	409.77	0.12
BBO	2.000056	2	2	409.78	409.83	409.79	0.016768	409.79	0.21
DE	2	2	2	409.77	410.59	409.8	0.14961	409.77	0.43
ACO	1	2	3	209.93	209.93	209.93	5.78E-14	209.93	0.24
SSA	0.136589	1.226449	13.14084	3.6619	3.7269	3.6884	0.020417	3.6885	0.003
SCA	0.138481	1.3	11.65668	3.6631	10.019	5.2877	1.2928	5.23	0.000
GWO	0.13916	1.3	11.8939	**3.6619**	3.6619	**3.6619**	**5.00E-06**	3.6619	0.000
nAOA	0.144392	1.271965	14.66017	**3.6619**	3.7554	**3.6819**	**0.022807**	3.6744	0.000
AOA	0.148662	1.3	15	4.0452	7.3439	6.1167	0.59017	6.1849	0.002

**Fig 9:**
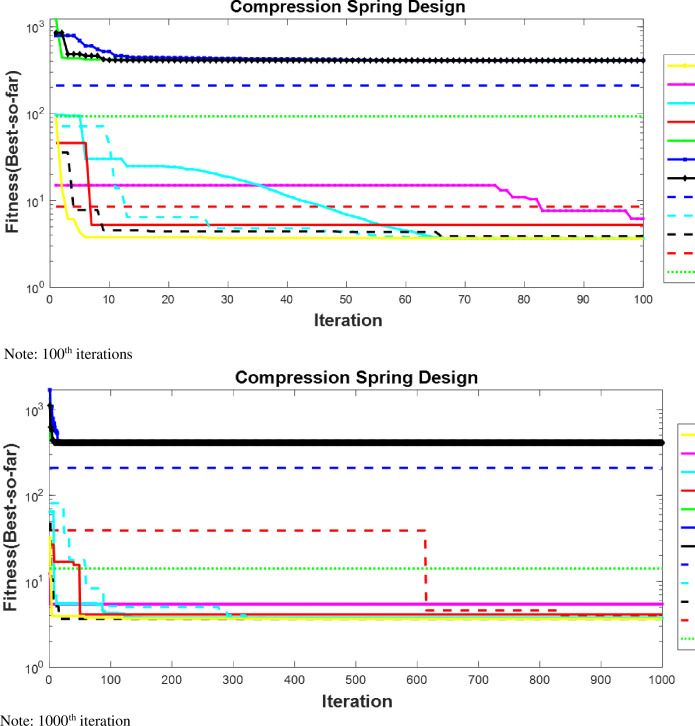
Convergence curves of CSD. Note: 100^th^ iterations. Note: 1000^th^ iteration.

The convergence curves for nAOA and 10 other algorithms used for the comparative analysis in the compression spring design are shown in Fig 9 at the 100th and 1000th iterations, in order to evaluate the algorithms’ behavior at both the early stage and later stage of the iterations. The curves for all the algorithms show irregular values at the start of the iterations; indicating that nAOA behaves similarly to the other algorithms. Since the algorithms were unable to find best results early in the iteration process, they searched the space for the optimal solution and were able to converge towards the best result and remain stable until the end of the optimization iterations. The dissimilarities in behavior of the curve at the different iteration phases show sensitivity to the initialization scheme used by the initial candidate solution. Moreover, we see the efficient performance of nAOA, AOA, GSA, GWO, SCA, and SSA because their curves lie together at the bottom of the figure. By contrast we note the sub-optimal performance of PSO, BBO, GA, and DE, indicated by curves lie together at the top of the figure because they show large cost function values.

#### 4.2.3. Pressure vessel design problem

**Fig 10:**
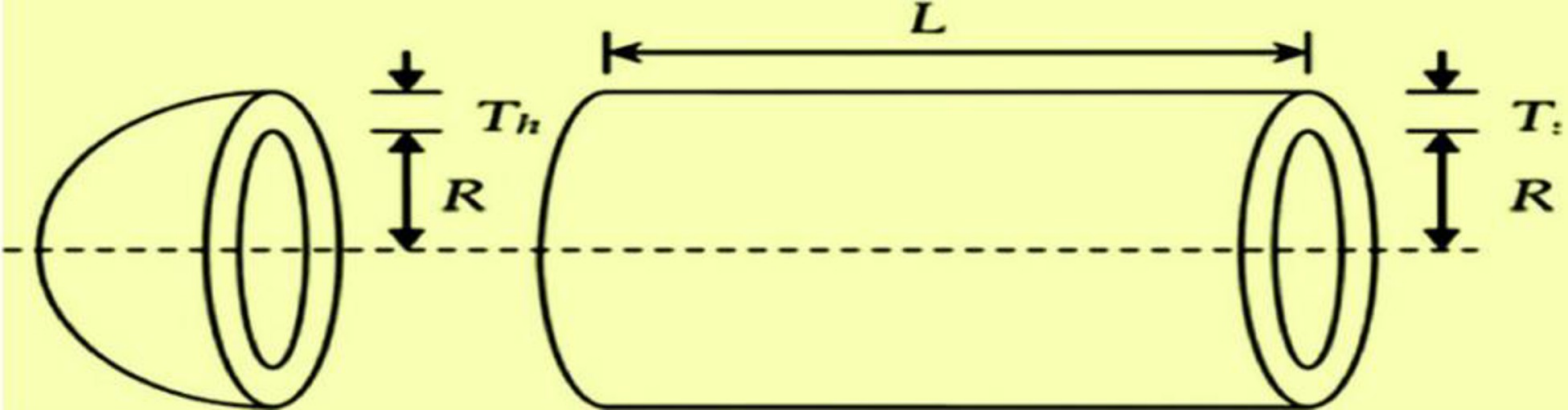
PVD problem [30].

A pressure vessel design model (PVD) is shown in Fig 10. The four decision variables are defined as follows: *x*1 is the thickness of the pressure vessel *T*_*s*_, *x*2 is the thickness of the head *T*_*h*_, *x*3 stands for the inner radius of the vessel *R*, and *x*4 is the length of the vessel barring head *L*.

The PVD can be formulated mathematically as follows [30]:

Given

$$l=[l_{1}l_{2}l_{3}l_{4}]=[T_{s}T_{h}RL],$$

$$Min\;f(\vec{l})=0.6224l_{1}l_{3}l_{4}1.781l_{2}l_{3}^{2}+3.1661l_{1}^{2}l_{4}+19.84l_{1}^{2}l_{3}$$ (7)

$$s_{1}(\vec{l})=-l_{1}+0.0193l_{3}\leq 0,$$

$$s_{2}(\vec{l})=-l_{3}+0.00954l_{3}\leq 0,$$

$$s_{3}(\vec{l})=-\pi l_{3}^{2}l_{4}-\frac{4}{3}\pi l_{3}^{3}+1296000\leq 0,$$

$$s_{4}(\vec{l})=l_{4}-240\leq 0.$$

The interval of the design variables are as follows:

$$0\leq l_{1}\leq 99,$$

$$0\leq l_{2}\leq 99,$$

$$10\leq l_{3}\leq 200,$$

$$10\leq l_{4}200.$$

The result for experiments for PVD is shown in Table 8, indicating the comparative analysis of all the optimization techniques for PVD problem; that is, nAOA and 10 other algorithms (GSA, PSO, BBO, DE, ACO, CPSOGSA, GWO, SCA, SSA, and AOA). The results show that nAOA outperformed the original AOA for the cost function of the PVD problem. Moreover, nAOA returned smaller values for mean and SD when compared with AOA, the same can be observed for the design variables *Ts*, *T*_*h*_, *R*, and *L*. However, again, the overall best performing algorithm in terms of the best, average, and standard deviation is GWO. Our proposed algorithm was, nevertheless, very competitive as it returned the same best cost value as GWO and came second to GWO for mean and standard deviation. The PSO, BBO, ACO, and DE have large values for the average, SD, and median indicating sub-optimal results for the cost. The Wilcoxon signed-rank test indicates that PSO, BBO, DE, and ACO simulation results are not statistically significant because they have p-values greater than 0.05. The results of SSA, SCA, GWO, nAOA, AOA, CPSOGSA, and GSA were significant because they have p-values less than 0.05.

**Table 8:** Results for experiment conducted for PVD.

	*Ts*	*Th*	*R*	*L*	Best	Worst	Average	SD	Median	p-values
CPSOGSA	1.09357	0	65.22523	10	2302.5	3638.6	4113.4	1326.8	3873.3	0.001
GSA	0.700037	0	49.29101	104.0716	3323.4	9287.2	3858.4	1441.6	3495	0.021
PSO	10	10	53.69781	71.47073	2.04E+05	2.04E+05	2.04E+05	1.8838	2.04E+05	0.2
BBO	10	10	50.94534	91.02183	2.04E+05	2.09E+05	2.05E+05	1258.2	2.05E+05	0.31
DE	10	10	53.71582	71.35088	2.04E+05	2.04E+05	2.04E+05	0.14598	2.04E+05	0.53
ACO	2	1	4	3	1.68E+16	1.68E+16	1.68E+16	8.1368	1.68E+16	0.324
SSA	1.090893	0	65.22699	10	2302.5	3638.6	3517.7	276.54	3626.5	0.002
SCA	1.090893	0	65.22699	10	2309.7	6065.6	4942.6	1733.4	6056.6	0.000
GWO	1.090893	0	65.22699	10	**2302.6**	6058.9	2556.8	**951.56**	2302.8	0.000
nAOA	1.076654	0.003068	65.10835	10.61673	**2302.5**	6078.2	3303.1	**519.16**	3567.6	0.000
AOA	0.212887	0.002212	44.39755	183.4564	3599	6270.8	4440.8	875.52	3997	0.001

**Fig 11:**
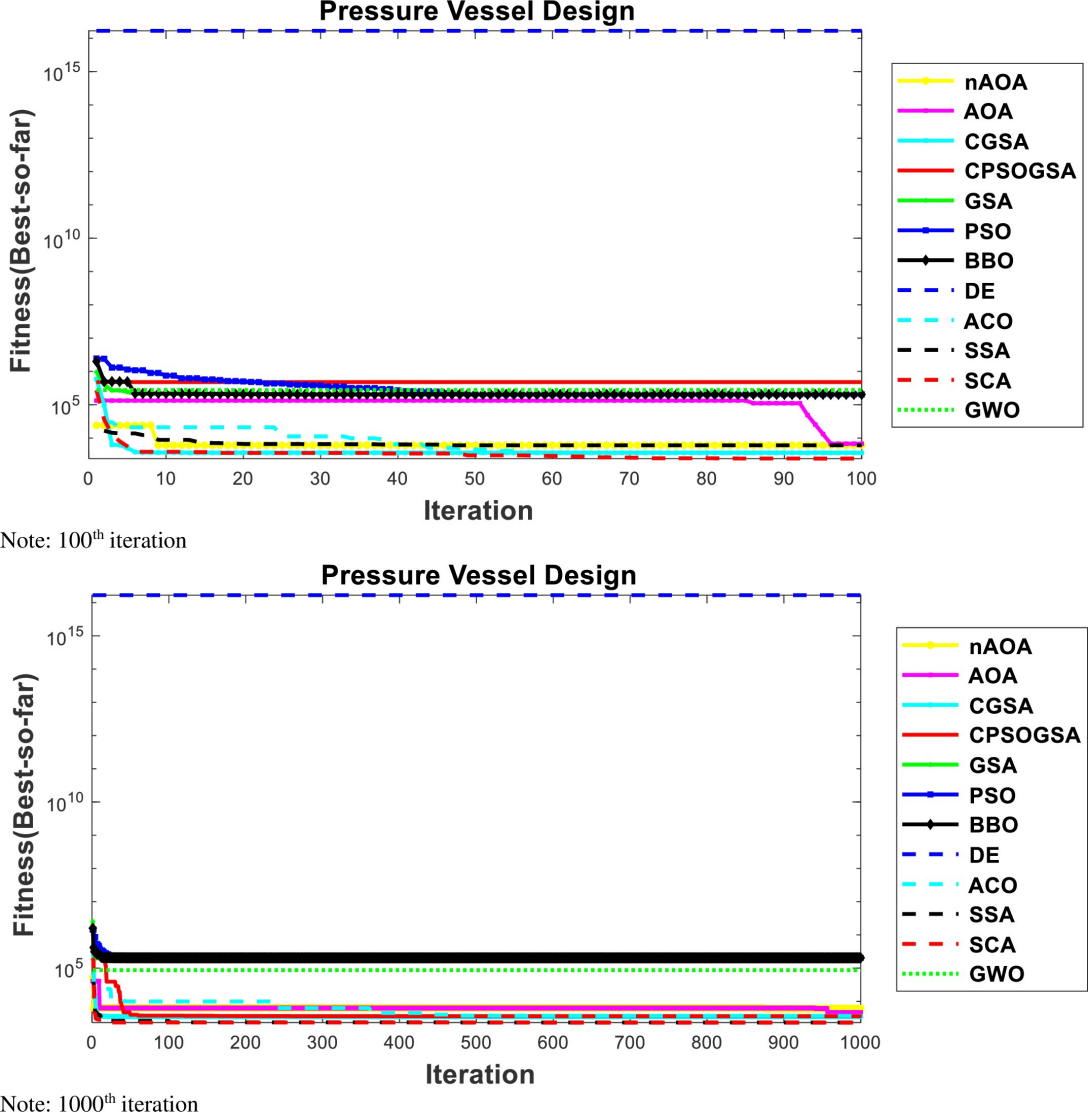
Convergence curves for PVD. Note: 100^th^ iteration. Note: 1000^th^ iteration.

The comparative analysis at the 100th and 1000th iterations for nAOA and 10 other algorithms used is shown by the convergence curves in Fig 11. The pairs of curves are shown to evaluate the algorithms’ behavior at the early and later stages of the iterations. The figure shows that nAOA has irregular values at the start of the iterations, as do the other comparative algorithms. The irregular values imply that the algorithms were unable to find best results early on, although as iterations progressed towards the later stages, the algorithms converged towards the best result and remained stable to the end of the optimization iterations. These results showing dissimilarities at the different iteration phases indicate the sensitivity of the algorithms to the initialization scheme used by the initial candidate solution. As can be seen, the convergence curves of nAOA, AOA, CGSA, GWO, SCA, SSA, and CPSOGSA all lie close to each other at the bottom of the figure because their values for the cost function are close. Then we note that the convergence curve for PSO, BBO, DE, and ACO also lie together, but above those for the rest of the algorithms, where their large values for the average, SD, and median indicate sub-optimal results for the cost. The efficient performance of nAOA, GSA, GWO, SCA, ACO, and SSA is indicated by their position at the bottom of the figure.

### 4.3. Overall simulation result’s discussion

This section gave overall analysis of the simulation results of all the 11 state-of-the-art algorithms used in our experiments. The algorithms are the proposed nAOA, the original AOA, and nine other state-of-the-art algorithms (CPSOGSA, GSA, PSO, BBO, DE, ACO, GWO, SCA, and SSA). The best performing algorithm overall is the GWO, because it returned best values for WBD, CSD, and PVD problems, while nAOA provides optimal values for the fitness function of WBD, CSD, and PVD (second only to GWO). In addition, the simulation results of the classical and composite (CEC 2020) benchmark functions convey that nAOA performed optimally, as can be seen from statistical results of average and SD values, which are very close to the global minimum.

The WBD problem results, shown in Table 6, indicate that nAOA, GWO, and CPSOGSA returned between 1.6957 and 1.6976 as best result, which is near the optimal cost value for WBD (0.69). Their respective average results are 1.7731, 1.6976, and 1.8545, which are also close to the global optimal for WBD. The GSA, AOA, and SSA have an average value of 2.8718, far from the best value (1.69). The algorithms PSO, BBO, GA, and DE all showed sub-optimal results.

Furthermore, Table 7 shows the result for the CSD problem. This set of results conveys that nAOA returned a best result of 3.6619, which is the same as that returned by GWO, SSA, and CPSOGSA. This result is better than that for GSA (3.7502), PSO (409.7), BBO (409.7), GA (409.7), DE (409.7), and ACO (209.9). The average results and standard deviations for nAOA, GWO, SSA, and CPSOGSA show that the algorithms could find near-optimal results early in the iteration process and quickly converge towards their best results. Nevertheless, these latter ’best’ results were still not as good as those for the nAOA, GWO, SSA, and CPSOGSA.

The results shown in Table 8 indicate that, for the PVD problem, nAOA, GWO, and CPSOGSA all returned 2302.6 as their best result. Their respective average results are 3303.1, 2556.8 and 4113.4. The performance of nAOA is second to only GWO, which had the best performance. The algorithms GSA, AOA, and SSA have average values between 3858.4 and 4440.8, which are not close to that returned by the best-performing algorithm. The algorithms PSO, BBO, GA, and DE showed sub-optimal results.

This overall analysis of the results of our experiments conveys that nAOA showed promising results for optimizing the classical and composite (CEC 2020) benchmark functions. It clearly outperformed the original AOA and was very competitive with the other 9 algorithms used. The same conclusion is seen for optimizing the fitness function and design parameters of the three mechanical engineering frameworks considered here, as indicated by comparing nAOA with the other participating algorithms. Another observation is that nAOA, AOA, GSA, GWO, SCA, SSA, and CPSOGSA provide better statistical results for the fitness function of the mechanical engineering design frameworks. However, the performance of PSO, BBO, GA, and ACO is suboptimal for all three engineering benchmarks.

## 5. Conclusion and future directions

In this paper, we proposed an improved nAOA algorithm that uses the high-density values that the natural logarithm and exponential operators can generate to enhance the exploratory ability of AOA. The addition and subtraction operators still carry out the exploitation phase. We tested the performance of the nAOA with 33 benchmark functions and three engineering design benchmarks. As a result, the nAOA has shown efficient performance for the WBD, CSD, and PVD (being second only to GWO).

This research has opened future research direction; it will be interesting to see how researchers can overcome the drawback of premature convergence and sensitivity in randomization. Researchers could use the stochasticity, ergodicity, and complex nonlinear motion properties of chaotic maps to overcome this drawback. In addition, nAOA could be applied to many other real-world problems, such as the economic load dispatch problems of electronic science. Furthermore, nAOA has considerable potential for hybridization with other state-of-the-art algorithms.
